# Tuberculin in Ophthalmic Practice

**Published:** 1915-04-03

**Authors:** Alfred C. Norman

**Affiliations:** Durham County and Sunderland Eye Infirmary, Sunderland.


### TUBERCULIN IN OPHTHALMIC PRACTICE.

### The Method Adopted and Results.

By ALFRED C. NORMAN, M.D., Ch.B. Edin., Durham County and Sunderland Eye Infirmary, Sunderland.

During the last three years at the Durham County and Sunderland Eye Infirmary we have made a practice of using tuberculin in the treatment of certain classes of ophthalmic cases, and the results have been so satisfactory that it seems worth while publishing particulars of the method we have adopted.

We give tuberculin as a matter of routine in all cases of phlyctenular keratitis and conjunctivitis; also in certain selected cases of blepharitis marginalis, when the patient presents evidence of the strumous diathesis, as well as in the definitely tuberculous lesions of the eye—i.e. those in which the tubercle bacillus can be demonstrated.

The *strumous* or *scrofulous* diathesis, better known as *scrofulosis*, presents a picture very familiar to all ophthalmic surgeons and to nearly all general practitioners. The patient has a generally unhealthy appearance, with more or less eczema of the face. The mucous membranes of the nose are inflamed and the anterior nares blocked with crusts, the end of the nose is congested and bulbous, and the upper lip swollen and thickened, giving to the face a typical expression. Enlarged tonsils, adenoids, and swollen cervical glands are frequently found, whilst tuberculous lesions of the bones and joints or pulmonary phthisis may or may not be present.

The majority of cases of scrofulosis are in children or young adults, and sooner or later most of them find their way to an eye hospital. If the patients seek treatment in the early stages they may have a few phlyctenules on the conjunctiva, and perhaps one on the cornea; but this stage is usually neglected, and they present themselves later with one or more small ulcers of the cornea, congestion, and intense photophobia. The worst cases of all come with extensive ulceration of the cornea, which has become vascularised, and in some cases the ulcer has actually perforated. At this stage the conjunctiva is very congested, the eyelids are swollen, and the margins of the lids are frequently thickened and ulcerated.

The tubercle bacillus can very rarely be demonstrated in the lesions of scrofulosis. What, then, is the nature of this disease, and why does it respond so readily to treatment with tuberculin?

### The Nature of Scrofulosis.

It seems to me that the solution of the problem lies in Virchow's definition of struma as "an abnormal vulnerability of the tissues." All these cases of struma or scrofulosis probably have somewhere in their bodies a latent (and often a hidden) *primary* focus of tuberculosis giving origin to toxic substances which, circulating with the blood-stream, produce an abnormal vulnerability of the tissues of the whole body. The tissues, especially the mucous membrane of the nose, the skin of the face, the conjunctiva, the lid margins, and the cornea, thus become an easy prey to some of the less virulent micro-organisms—probably belonging to the stapyhlococcal group. The action of tuberculin is to stimulate the production of protective anti-bodies which, reacting upon the *primary* tuberculous focus, diminish the toxæmia and increase the general resistance of the tissues. Thus the lesions in the eye and elsewhere tend to disappear spontaneously or become remarkably amenable to local treatment of the simplest nature.

In many of the eye cases one is given a history of a recent attack of measles; in fact this malady is far too frequently blamed for the whole condition. Probably, in most instances, the toxæmia of measles is simply the last straw which, added to the toxæmia of an already existing tuberculous focus, ensures the defeat of the tissues. In other cases it may be that the, as yet undiscovered, organism of measles is actually present in the eye lesions, having obtained a hold as a result of the child's scrofulous tendency. Whatever the explanation, these cases respond to tuberculin quite as readily as the ordinary scrofulous ones.

### Precautions in the Use of Tuberculin.

In giving tuberculin to ophthalmic cases it is of the utmost importance to avoid producing an exacerbation of the eye symptoms. Since the tubercle bacillus is generally not present in the eye lesions, one might be inclined to wonder how an excessive dose of tuberculin could produce such an exacerbation, but the fact remains that it does, and the explanation is not far to seek. Too large a dose of tuberculin produces an excessive reaction in the aforementioned *primary* focus which in its turn contributes more toxic substances to the blood stream, and thus further reduces the resistance of the body tissues. In consequence, the organisms at work in the eye lesions gain a fresh lease of life. The reaction produced in the *primary* tuberculous focus being called a *focal* reaction; that in the eye lesions might be termed a *secondary focal* reaction. The term *local* reaction is used for the reaction at the site of injection and *general* reaction for the systemic disturbance (pyrexia, etc.) which sometimes follows an excessive dose of tuberculin.

The system of dosage given below has been ultimately arrived at by the writer with the object of producing a gradually increasing immunity without at any stage giving rise to a focal reaction.

The method of preparing diluted tuberculin is both simple and inexpensive, so that no one need hesitate to adopt tuberculin treatment on account of its supposed trouble or costliness. The doses are expressed in minims and fractions of a milligramme instead of by the customary decimals, which frequently convey so little to the British mind. The writer finds it satisfactory to prepare a fresh supply of tuberculin about once a week.

Method of Preparing Tuberculin at the Sunderland Eye Infirmary.

**Apparatus Required.**—A hypodermic syringe, a 100-minim glass measure, four two-drachm stoppered phials, indelibly labelled "A," "B," "C," and "D" respectively, and a 4-oz. stoppered bottle containing a 5 per cent. solution of glycerine in distilled water. All these, including the bottle of glycerine, are boiled in an open steriliser immediately before the tuberculin is prepared. The tuberculin is purchased from Messrs. Allen and Hanburys, in sterile azoules each containing 1 mg. of T.R. The contents of one azoule (generally about twenty minims) are withdrawn by means of the hypodermic syringe, and are diluted to 100 minims with the 5 per cent. glycerine solution. This solution, now containing 1 mg. of T.R. in 100 minims, is placed in bottle "A."* Ten minims of the solution are withdrawn from bottle "A," diluted to 100 minims, and placed in bottle "B." Ten minims from bottle "B" are diluted to 100 minims and placed in bottle "C." Ten minims from bottle "C" are diluted to 100 minims and placed in bottle "D."

From these bottles any dose can be withdrawn by means of a sterile syringe and injected into the patient as required. At the Sunderland Eye Infirmary we simply order so many minims from bottle "A," "B," "C," or "D," as the case may be, and the injections are given by a specially trained nurse. Each patient is given an injection once a week, starting with 10 minims from bottle "D" (1-10.000th mg. T.R.), and working up to 20 minims from bottle "A" (1-5th mg. T.R.) by thirteen weekly increments as follows:—

| | | Mg. T.R. |
|---|---|---|
| 10 minims | from bottle | D = 1-10.000th |
| 20 | „ | D = 1-5.000th |
| 4 | „ | C = 1-2.500th |
| 8 | „ | C = 1-1.250th |
| 10 | „ | C = 1-1,000th |
| 20 | „ | C = 1-500th |
| 4 | „ | B = 1-250th |
| 8 | „ | B = 1-125th |
| 10 | „ | B = 1-100th |
| 20 | „ | B = 1-50th |
| 4 | „ | A = 1-25th |
| 8 | „ | A = 2-25ths |
| 10 | „ | A = 1-10th |
| 20 | „ | A = 1-5th |

Table of Doses Recommended for Injection at Intervals of One Week.

Very occasionally a slight reaction is produced at the seat of the eye lesion, and when this occurs the dose of T.R. is repeated the next week instead of being increased.

The injections are given subcutaneously in the deltoid region, the skin having been previously cleansed with ether soap. There is frequently a small local reaction, which should be specially observed after the first injection as it is of diagnostic importance. This reaction is never severe; in fact, out of several thousand injections there has been no suggestion of suppuration—a result which I attribute largely to the very careful technique of the nurse who gives the injections—Miss F. Simon.

We have had no instance of a general reaction The improvement, under tuberculin treatment, in the general condition of these scrofulous children is very remarkable, and the eye lesions clear up in a miraculous way with the aid of simple local treatment, such as atropine and yellow oxide of mercury ointment. A few of the worst cases are kept in bed for purposes of observation and control, but the majority are treated as out-patients.

It is not generally recognised that a very large amount of defective vision in this country is caused by phlyctenular (scrofulous) keratitis. The pity of it is that it is so frequently neglected, for there is no disease more amenable to treatment in its early stages. Once the cornea has become deeply ulcerated, however, a nebula or a leucoma will remain for the rest of the child's life, the immediate result of which is more or less irregular astigmatism and the ultimate result, frequently, myopia. In very young children the more involved eye is likely to become amblyopic from disuse.

Under tuberculin treatment the process of ulceration is arrested very much more quickly than by ordinary methods alone, and the resulting area of corneal opacity is correspondingly diminished. Another advantage is a remarkable freedom from relapse, but to ensure this we find it necessary to give the full course of fourteen injections in all cases. A short while ago, on account of the internal arrangements of the hospital, we were obliged to discontinue tuberculin treatment for about six weeks, and during that time the writer was greatly struck by the comparatively slow progress made by all fresh cases of phlyctenular keratitis.

* Where large quantities are used an original phial, containing 10 mg. of T.R., can be obtained. This must be diluted to 1,000 minims and placed in a bottle labelled "A." The other bottles can be prepared from this as required.

